# Functional and Structural Network Impairment in Childhood Frontal Lobe Epilepsy

**DOI:** 10.1371/journal.pone.0090068

**Published:** 2014-03-04

**Authors:** Maarten J. Vaessen, Jacobus F. A. Jansen, Hilde M. H. Braakman, Paul A. M. Hofman, Anton De Louw, Albert P. Aldenkamp, Walter H. Backes

**Affiliations:** 1 Department of Radiology, Maastricht University Medical Centre, Maastricht, the Netherlands; 2 Department of Research and Development, Epilepsy Centre Kempenhaeghe, Heeze, the Netherlands; 3 School for Mental Health and Neuroscience, Maastricht University Medical Centre, Maastricht, the Netherlands; 4 Department of Neurology, Maastricht University Medical Centre, Maastricht, the Netherlands; Wake Forest School of Medicine, United States of America

## Abstract

In childhood frontal lobe epilepsy (FLE), cognitive impairment and educational underachievement are serious, well-known co-morbidities. The broad scale of affected cognitive domains suggests wide-spread network disturbances that not only involves, but also extends beyond the frontal lobe. In this study we have investigated whole brain connectional properties of children with FLE in relation to their cognitive impairment and compared them with healthy controls. Functional connectivity (FC) of the networks was derived from dynamic fluctuations of resting state fMRI and structural connectivity (SC) was obtained from fiber tractograms of diffusion weighted MRI. The whole brain network was characterized with graph theoretical metrics and decomposed into modules. Subsequently, the graph metrics and the connectivity within and between modules were related to cognitive performance. Functional network disturbances in FLE were related to increased clustering, increased path length, and stronger modularity compared to healthy controls, which was accompanied by stronger within- and weaker between-module functional connectivity. Although structural path length and clustering appeared normal in children with FLE, structural modularity increased with stronger cognitive impairment. It is concluded that decreased coupling between large-scale functional network modules is a hallmark for impaired cognition in childhood FLE.

## Introduction

Frontal lobe epilepsy (FLE) is considered to be, after temporal lobe epilepsy, the second most common type of the localization-related (partial) epilepsies of childhood and accounts for 20–30% of partial epilepsies [1]. Pediatric FLE, even when cryptogenic in nature, is frequently complicated by the impairment of a broad range of cognitive problems, behavioral disturbances, and therapy resistance [2]. The fact that all these complications occur at a young age is troublesome. In childhood the brain is at its most vulnerable state and neurologic disturbances such as FLE can have an impact on brain maturation and the development of cognitive skills, with potentially severe consequences for school performance [3].

The broad range of affected cognitive domains suggests a global network disturbance, rather than perturbations of localized individual processes. Disturbances in network organization can be assessed by connectome analysis, which comprises the mapping of the nodes and connections of the human cerebral network [4]. Cerebral connectivity may either be of functional or structural nature. Functional connectivity (FC) can be measured by correlating blood-oxygen-dependent oxygenation (BOLD) related dynamic fluctuations of gray matter activity between different brain regions [5] and structural connectivity (SC) can be obtained by tracing fiber bundles through the white matter with fiber tractography [6].

Resting state functional MRI (RS-fMRI) enables the investigation of the intrinsic functional organization of the brain and is typically measured by the temporal correlation of neuronal activity-induced signal variations of anatomically different brain regions [7], [8]. Previous studies have demonstrated disruptions in functional networks of adult and pediatric epilepsy patients [9], [10], [11], which have also been related to cognitive and epilepsy variables [12], [13], [14], [15], [16]. Structural network abnormalities have also been implicated in adult epilepsy patients [17], [18], [19]

Modeling the brain as one system of nodes (brain regions) and edges (connections) allows a direct comparison of SC and FC, because the organization of nodes and edges can be derived from both functional and structural imaging data. Apart from correlating the functional and structural connection strengths of individual edges, one can also explore and relate the topology of SC and FC networks in terms of graph theoretical measures. Graph theoretical analysis has the advantage that topological properties of the whole brain network can be captured in a few summary measures that describe the amount of segregation and integration among brain regions [20], [21].

Although changes in either FC or SC are interdependent [22], the relation between FC and SC is likely complex [23]. The white matter connectivity provides a physical substrate that possibly constraints the functional connectivity between different brain regions [24], [25], [26]. Several studies have indicated that SC is predictive for FC, while FC is not predictive for SC, across healthy human brain networks [25], [27]. The SC-FC relation increases in strength during normal development [24] and might be disrupted in the diseased brain [28], [29]. However, it is unclear whether abnormalities in the dependency between FC and SC are present in FLE and can explain cognitive impairment.

Previously, it was observed that the neuronal basis for cognitive deficits in FLE reside in the interaction between large-scale functional brain sub networks, the so-called modules [30]. The whole brain network can be divided into sub networks by modular decomposition (i.e. community structure) methods [31], and therefore, this method provides the opportunity to investigate the connectional properties of the different large-scale sub networks. The modular structure of the brain network is thought to be important for cognitive abilities, as increases in coherent activity between functional systems might facilitate adaptive behavior and the integration of information [32], [33]. In FLE, the cognitive pathology might be reflected through reductions in coupling between sub networks, which can either be of functional or structural origin or both. This nature of network abnormalities has not been addressed explicitly yet, however several studies have indicated that functional network abnormalities are present in adults with epilepsy [15], [29] as well as children with FLE [16], [34]. Furthermore, a recent study showed that structural networks were affected in a related childhood epilepsy [35].

In this study we investigate functional as well as structural whole brain networks in children with FLE. We explore whether abnormalities in graph theoretical measures are present for both the functional and structural networks and correlate these with the cognitive impairment. We hypothesize that differences in whole brain graph theoretical measures can be explained by differences in connectivity between and within large-scale modules. Moreover, the coupling between SC and FC connectivity was compared between children with FLE and healthy controls and correlated with the cognitive impairment.

## Methods

### Participants

Children with FLE were selected from our reference clinical database and were actively contacted. Inclusion criteria were: a clinically confirmed cryptogenic (i.e., based on clinical presentation, EEG and MRI findings, presumed to be symptomatic, but with unknown etiology) localization-related epilepsy with an epileptic focus in the frontal lobe, aged between 8 and 13 years, no other disease that could cause cognitive impairment, and no history of brain injury. Healthy age-matched controls were recruited by advertisements in local newspapers. No history of brain injury or cognitive problems was allowed and controls were visiting regular education. This study was approved by the Institutional Review Board of the Maastricht University Medical Center. All subjects and parents gave written informed consent.

### Neuropsychological testing

Cognitive performance was measured using a computerized visual searching task (CVST) [36]. This task consists of finding the right grid pattern that matches the one in the centre of a screen surrounded by 24 other grid patterns. The task is used to assess central information processing speed and perceptual strategies and is considered to be an assessment of frontal lobe function. A detailed description of this task can be found in [30], [36]. By determining the average CVST searching time (reaction time) and the number of correct and incorrect responses during the task, an age-corrected cognitive performance score was generated (i.e. the decile score). After grouping these scores into numbers from 1 (worst score) to 10 (best score), the 3 worst performance scores (1, 2 or 3) were considered a manifestation of impaired cognitive performance, while higher scores (≥4) were considered normal. We compared the entire patient group (EP) and the cognitively impaired patient group (IP) with the healthy control group.

### MRI acquisition

MRI was performed on a 3.0-Tesla unit equipped with an 8-channel head coil (Philips Achieva, Philips Medical Systems, Best, The Netherlands). Functional MRI data were acquired using a whole-brain single-shot multi-slice echo-planar imaging (EPI) sequence sensitive to the blood-oxygen-level-dependent (BOLD) effect, with TR 2 s, TE 35 ms, flip angle 90°, FOV 200×200 mm, matrix 112×108, pixel size 2×2 mm^2^, 32 contiguous 4-mm thick slices per volume, 195 volumes per acquisition, and an parallel imaging acceleration factor of 1.5 (Sensitivity Encoding).

Diffusion weighted MRI (DWI) was acquired at a pixel size of 2×2 mm^2^, slice thickness 2 mm, and a b-value of 1200 s/mm^2^. An echo planar imaging sequence was used with TE 72 ms, TR 6584 ms, and parallel imaging acceleration factor of 2. A set of 61 gradient directions was used, optimized via electrostatic repulsion to ensure homogenous distribution over the sphere [37]. In addition, a single non-diffusion weighted scan (b0-scan) was obtained. The DWI acquisition time was 8 minutes.

For anatomic reference, a T1-weigthed 3D spoiled fast gradient echo pulse sequence was acquired with the following parameters: TR 8.1 ms, TE 3.7 ms, flip angle 8°, field of view (FOV) 256×256×180 mm^3^, and voxel size 1×1×1 mm^3^.

### Inclusion

Subjects were excluded when head movements exceeded 1.5 mm/s or 1.5 degrees/s in at least one direction. Data of nine patients were excluded from further analysis because of movement related artifacts (n = 6; 2 controls, 4 patients), EPI artifacts (n = 3; 2 controls, 1 patient). From the DWI data, five subjects were excluded from further analysis due to EPI artifacts (n = 1 controls, n = 4 patients). For final analysis, the study population consisted of 26 patients and 36 healthy controls.

Six patients did not complete the neuropsychological assessment and thus had no CVST scores. These patients are included in the group analysis, but not in the correlation analysis. In total, 9 FLE patients had a decile score below 4 and were considered cognitively impaired. Mean CVST reaction time was significantly higher in the patient group compared to the healthy control group (controls: 7.5±6.4 s (mean ±SD), patients: 23.6±9.9 s, p<0.006). The age of the patient group (132 months) and the control group (125 months) was not significantly different (p<0.1), nor was the ratio between male/female different between the groups (χ^2^-test: p<0.17).

### Network construction

#### Anatomical parcellation

Freesurfer (Martinos Center of Biomedical Imaging, Boston, US) software was used to segment the T1 images of each subject into 82 cortical and subcortical regions. Freesurfer uses a surface based alignment procedure, which might be more accurate than a volume based alignment of a cortical atlas [38]. Freesurfer applies a standardized processing pipeline to the T1 image including skull stripping, segmentation of white and grey matter and CSF.

The Freesurfer cortical regions were further refined into a larger number of smaller regions. We started by dividing each region from the standard Freesurfer template into two more or less equally sized regions by principal component analysis. This segmentation was performed in the spherical surface coordinates. The cortical surface of each hemisphere can be modeled as the surface of a sphere; each point on the cortical surface can be related to a point on the sphere which is defined by its longitude and latitude. The first principal component (a 2D vector), together with the center of gravity of the cortex point within the region (a 2D point), defines a line in 2D space which divides the region into two sub regions according to the maximum spatial variance of the region (e.g. a “stretched” region will be divided along its main longitudinal axis). Regions were subsequently subdivided with the criterion that a division must not yield a sub region with a size smaller than 1200 cortical points. The final result was a parcellation of 97 regions with comparable sizes (of at least 1200 cortex points) per hemisphere defined in the Freesurfer standard space. The regions were converted from the Freesurfer standard space spherical format to the cortex surface model and subsequently to the T1 volume format of each individual by standard Freesurfer routines. The subcortical regions were used in their original Freesurfer format and not further divided.

The cortical and subcortical parcellation of each individual in native T1 space was transformed to the native DWI or fMRI space by applying a rigid body transformation computed with the FSL FLIRT tool [39]. The transformations from Freesurfer standard space to T1 space and from T1 space to DWI or fMRI space can result in the loss of several regions by partial volume effects. Therefore, only regions that were present in all participants after transformation to DWI and fMRI space were used in the final parcellation. This resulted in the same parcellation with 205 bi-lateral regions for each subject (2×95 cortical regions and 15 subcortical regions) in the individual T1, DWI and fMRI spaces.

#### Functional network construction

The BOLD images were corrected for motion artifacts using SPM5 (Wellcome Trust Centre for Neuroimaging, UCL, London, UK) software. The images were then high-pass filtered with a σ of 25 scans (0.02 Hz) and spatially smoothed (σ = 1.7 mm) using FSL 4.1.7 (Oxford University, Oxford, UK) software. Subsequently, the CSF, whole brain signal time course and motion parameters were removed from the images using standard linear regression. The resulting residual time series of the cerebrum were used for further analysis. Lastly, the images were low-pass filtered (σ = 2 s or 0.5 Hz, i.e. 1 dynamic scan interval) to remove the detrimental effects of high-frequency noise components. Using Matlab (The MathWorks Inc., Natick, US; version 7.6.0), the Pearson's linear correlation coefficient was calculated between the region-averaged time-series of all pairs of Freesurfer regions. In this way, a 205×205 connectivity matrix was calculated for each subject.

The removal of the whole brain average time series signal tends to shift the correlation distribution to have a mean value that is close to zero, thereby creating negative correlations even if no such correlations are initially present in the data [40]. Low (absolute) correlation coefficients could adversely affect the results as they may either represent physiologically relevant signal or just noise. Therefore, only positive correlations were used for further graph theoretical analysis.

#### Structural network construction

Each data set was spatially co-registered to the b = 0 image with an affine transformation to correct for head motion and eddy-current distortions utilizing CATNAP (Co-registration, Adjustment, and Tensor-solving, a Nicely Automated Program, version 1.3) software [41]. The set of gradient vectors was adjusted according to the rotation of the individual images.

All DWI analyses, the tractography and tract segmentations were performed using the MRtrix software package [42]. Diffusion tensor (DT) fits were performed to calculate FA and ADC maps. In addition, fiber orientation distributions (FODs), representing local fiber orientation, were estimated using constrained spherical deconvolution (CSD) [42]. The CSD response function was estimated from data with high FA voxels values (FA>0.7). Slice drop-outs are a common phenomenon in DW-EPI, especially in the presence of head motion [6], [43]. To reduce the effects of corrupted slices on FOD estimation, a method was developed in which corrupted slices were automatically detected and removed from the data. Subsequently, FOD's were estimated per slice with a slice-specific gradient set (i.e. without the directions corresponding to the removed slices). On average 46 (out of 3660. i.e. approx. 1.3%) slices were corrupted per subject. The number of corrupted slices did not differ between the groups.

Within the white matter, five million evenly distributed seeds were placed and a streamline was started from each seed. Subsequently, for each pair of regions from the anatomical atlas, the subset of tracts connecting these two regions were identified from the set of tracts of the whole brain tractogram Connection weights were determined by calculating the tract volume of the voxels traversed by the streamlines of the connection, divided by the total intracranial volume [44]. As an additional noise filter, voxels that were traversed by fewer than 2 tracts were eliminated from the analysis.

### Network analysis

#### Mean network connectivity

The mean connection strengths of the connection matrices were investigated for group differences and correlated with age. The SC networks were weighted with relative tract volume, mean tract FA, and mean tract ADC values. The Fisher-z transformed correlation values were used for comparison of FC between groups.

#### Network thresholding

From this point in the analysis pipeline the FC and SC matrices are treated similarly, both are an abstract representation of the human connectome and no further distinctions in graph analysis methods are made. Each subject's brain graph was thresholded to create graphs with an equal number of nodes and edges across subjects [15], [45]. This was achieved by selecting the *T_k_* connections with the highest edge weight and removing all other connections. The edge weight for the SC matrices was the relative tract volume and for the FC matrices the correlation strength. The threshold value *T_k_* was expressed as a sparsity value relating the number of edges maintained in the network to (twice) the total number of edges possible (N^2^−N). Let *T_k_* be the number of edges maintained in the network, then the sparsity (s) is defined as:
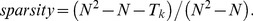
(1)In the remainder of this article, results will either be presented for a particular sparsity value or as a function of sparsity. For each individual FC or SC matrix, connection weights were scaled by the mean of this matrix as differences in mean weight can potentially influence weighted network metrics [46].

#### Network characteristics

For each subject, values of network measures were calculated from the individual SC or FC matrices. We included 3 network measures: characteristic path length (L), clustering coefficient (C), and the modularity coefficient, using algorithms implemented in the Brain Connectivity Toolbox [47]. A detailed description of path length and clustering can be found elsewhere [47]. It is important to note here that these metrics were computed for each individual participant, while the below described modularity *organization* was computed for the connectivity matrices averaged over the entire study population.

Modularity quantifies the degree to which a brain network is organized in isolated sub networks (i.e. the modules). The more isolated the sub networks are, the higher the modularity coefficient. We used the algorithm developed by Newman et al. [31] to quantify the modularity of the brain. With this algorithm the brain was automatically subdivided into a number of modules (i.e. groups of connected nodes) with maximal connection strength within and minimal connection strength between the modules, creating a so-called “optimal community structure” (OCS) of the brain. To avoid effects of differently organized modules in patients and controls, the within- and between-module connectivity was determined from the connectivity matrix that comprised the mean of entire study population, thus the combination of patients and controls.

#### Analysis of within- and between-module connectivity

To assess the potential differences in within- and between-module connectivity, the modular organization of the FC was calculated by applying the modularity algorithm to the FC matrix averaged over all subjects. Next, each edge was classified as either between-module (the edge connects nodes of two different modules) or within-module (the edge connects nodes of the same module). Connection strengths of the within- and between-module edges from the FC and SC modules were averaged. The within-module connections were assessed twice: as the aggregate over all modules and for each module separately. The weak and negative edges of the FC matrices might contain relevant information on between-module connectivity, therefore the un-thresholded FC and SC matrices were used.

### Statistical analysis

Between-group effects (for the EP, IP and healthy control group) in terms of network measures and connection strengths were assessed by two-sample Student's *t*-tests. Pearson's (linear) correlation coefficients (r) were calculated between cognitive performance (CVST reaction time), age, connection strengths and network measures. This analysis was performed for the EP group and control group, separately.

After the coupling between the FC and SC edge strengths was calculated by a correlation analysis for each subject, the individual FC-SC coupling values were associated with CVST scores and age. Significance was assessed at p<0.05 and trends at p<0.1.

## Results

### Network connectivity

#### Functional connectivity

The mean functional connectivity value (i.e. Fisher-z transformed time series correlations) over all connections did not significantly differ between the EP, IP and healthy control groups. Mean FC was not significantly correlated with age or with CVST scores.

#### Structural connectivity

The structural connectivity (i.e. relative tract volume over all connections) of the IP group (9.5·10^−4^±0.5·10^−4^, p<0.07), but not the EP group (8.9·10^−4^±0.4·10^−4^, n.s.), showed a trend of higher mean connectivity values than the control group (8.5·10^−4^±0.1·10^−4^). Mean structural connectivity was not correlated with CVST score or age.

Mean FA was not different between the groups, but increased with age in both the control (r = 0.32, p<0.05) and EP group (r = 0.44, p<0.02). Mean ADC was also not different between the groups and did not significantly correlate with age in the control group, while a negative correlation was found in the EP group (r = −0.47, p<0.01).

### Network topology

#### Functional connectivity

**Figure 1:**
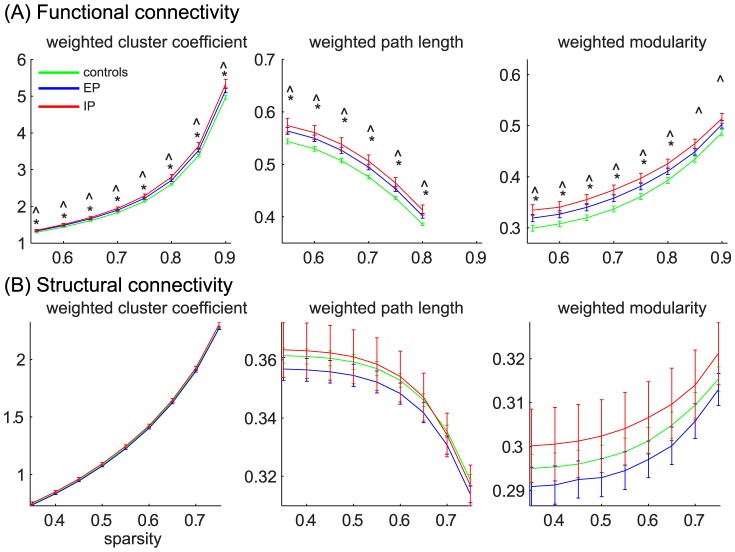
Network metrics for functional (A) and structural (B) connectivity as a function of sparsity. The networks measures for the control group (green), the entire patient group (EP, blue) and the impaired patient group (IP, red) as a function of sparsity. (A) Network parameters for the functional networks. (B) Network parameters for the structural networks. Symbols for statistical comparison: *: p<0.05 for EP versus control group, ∧: p<0.05 for IP versus control group.

The cluster coefficient was significantly higher for both the EP and IP groups compared to the healthy control group over the entire tested sparsity range (0.55–0.90). The path length was significantly higher for the EP and IP groups compared to the control group over the sparsity range 0.55–0.80. The modularity coefficient was significantly higher for the IP group compared to the control group over the entire sparsity range, while the EP group had a significantly higher modularity over the sparsity range 0.55–0.80 (Fig. 1A).

Neither the cluster coefficient, path length, nor modularity coefficient of the functional network were significantly correlated with CVST score or age for the control or EP group.

#### Structural connectivity

Although the cluster coefficient, path length, and modularity showed slightly higher values for the IP group compared to the control and EP groups, none of these differences were significant. The EP group also did not display significant differences compared to the control group (Fig. 1B).

In the EP group there was a negative correlation trend between the cluster coefficient and age (mean r = −0.37, p = 0.06) and a negative correlation between modularity and age (mean r = −0.40, mean p = 0.04). In the control group, the path length showed a trend of positive correlation with CVST scores (mean r over entire sparsity range = 0.29, p = 0.09). Modularity scores increased with CVST scores in the EP group (mean r = 0.51, p = 0.02).

### Modular organization

**Figure 2:**
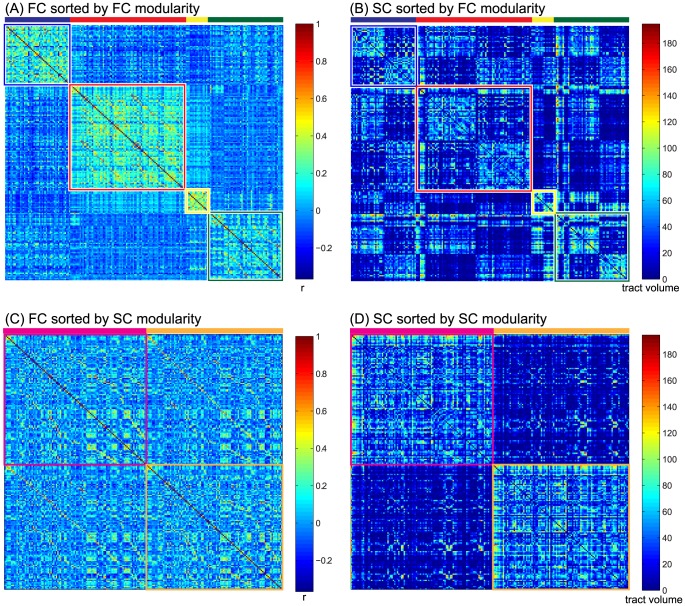
Modular organization. Group average connection matrices sorted by module. (A) Functional connectivity. Colored rectangles indicate the modules. High within-module connectivity is clearly visible by the higher values (more hot colors), while between-module connectivity is more sparse (more cold colors). (B) Structural connectivity sorted by functional modules. The functional modules are organized bi-laterally, while the SC has strong inter-hemispheric connectivity and low intra-hemispheric connectivity clearly visible in the block patterns. (C) The FC matrix sorted by the modular organization derived from the SC. The two found SC modules are basically the left and right hemisphere. From the FC it is visible that strong inter-hemispheric connections are present within the two modules. (D) The SC sorted by SC modularity. Strong intra-hemispheric connections are visible, while inter-hemispheric connections (and thus between-module connections) are weaker.

The modularity algorithm determined four modules from the averaged FC matrix over all subjects (Fig. 2A). The spatial organization of module 1 highly resembles the default mode network (DMN) [48], with regions in the frontal, temporal and parietal lobes. The second module consisted of frontal and subcortical regions. The relatively small third module was centered in the occipital lobe. Module 4 was distributed over frontal, temporal and occipital regions. All modules were highly symmetric with respect to the interhemispheric fissure. When the structural connections were ordered according to the organization of the FC modularity matrix, the structural organization of the modules revealed bilateral structural sub networks (Fig. 2A and 2B).

For the SC matrix the modularity algorithm determined only two modules, which were highly symmetric over the two hemispheres. After functional connections were ordered according to these SC modules, no further sub organization became evident (Fig. 2C and 2D).

#### Modular connectivity

FC (Fisher-z transformed correlation values) and SC (relative tract volumes) values were classified as between-module, within-module averaged over all modules (i.e. aggregated within-module) and individual within-module connections. These connection values are listed per group in Table 1.

**Table 1:** The modularity measures derived from the functional connectivity (FC) and structural connectivity (SC) matrices of the entire patient (EP) group, the cognitively impaired patient (IP) group, and the healthy controls.

	Controls (mean±SEM)	EP (mean±SEM)	*p-value (C-EP)*	IP (mean±SEM)	*p-value (C-IP)*
**FC**					
**BT**	−0.026±0.003	−0.033±0.004	*0.06*	−0.041±0.005	***0.02***
**WI**	0.245±0.005	0.260±0.008	*0.08*	0.265±0.011	*0.10*
**WI1**	0.284±0.021	0.299±0.010	*n.s.*	0.313±0.024	*n.s.*
**WI2**	0.244±0.005	0.255±0.014	*n.s.*	0.258±0.013	*n.s.*
**WI3**	0.606±0.007	0.643±0.029	*n.s.*	0.700±0.057	*0.07*
**WI4**	0.176±0.005	0.197±0.009	*0.07*	0.196±0.009	*0.07*
**SC** (*10^−4^)					
**BT**	7.8±0.1	7.9±0.1	*n.s.*	8.3±0.1	*0.07*
**WI**	9.9±0.2	10.2±0.2	*n.s.*	11.0±0.4	*0.08*
**WI1**	9.70±0.2	10.0±0.3	*n.s.*	11.0±0.6	*n.s.*
**WI2**	8.0±0.2	8.2±0.2	*n.s.*	7.9±0.3	*n.s.*
**WI3**	15.0±0.4	14.7±0.4	*n.s.*	16.0±0.8	*n.s.*
**WI4**	13.0±0.3	14.0±0.5	*n.s.*	15.0±0.1	***0.01***

The modularity measures comprise the between-module (BT) and within module connectivity values; the latter averaged over all four functional modules (WI) and per functional module (WI1…4).

#### Between-module connectivity

**Figure 3:**
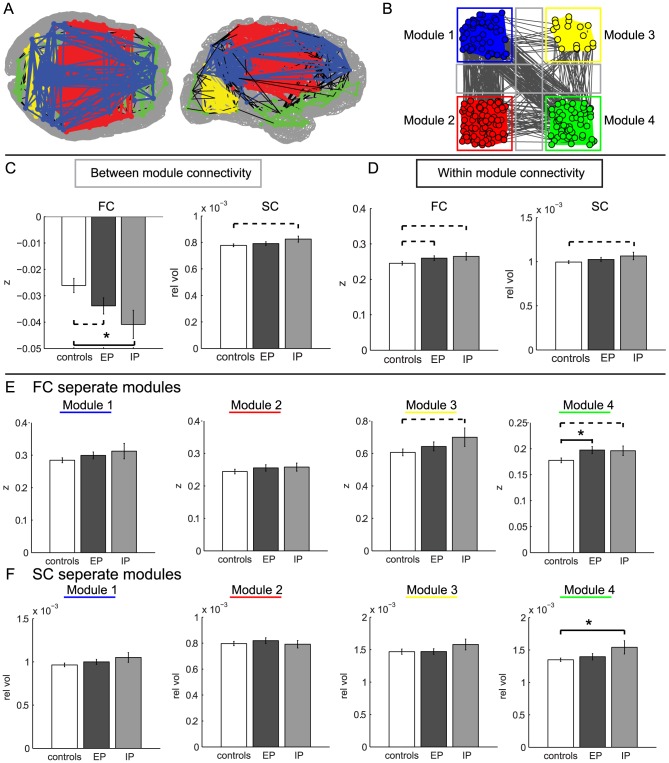
Within- and between-module connectivity for FC and SC. (A) Modular organization of FC. Within-module connections are colored as in Figure 2A. (B) An alternative presentation of the modular organization. The nodes of each separate module are depicted spatially segregated. The gray lines indicate the between-module connections. (C) All between-module connection strengths (gray lines in B) for FC and SC were averaged and compared between the different groups. (D) The within-module connections over all four modules were also averaged for the different groups and compared. (F) FC mean within-module connection strengths are compared between the groups for the four different modules. (E) Within-module SC. Bars display the mean+SEM.

The between-module FC was lower in the IP group (p<0.017) and the EP group (p<0.06) compared to the control group. A trend for higher between-module SC was found in the IP group compared to the healthy control group (p = 0.07), while no differences were found between the EP and control group (Fig. 3C). No significant correlations were found between CVST score or age and FC or SC between-module connectivity.

#### Aggregated within-module connectivity

A trend for higher within-module FC values was observed for the IP group (p = 0.095) and the EP group (p = 0.08) compared to the control group. The SC within-module connection strengths displayed a trend for higher values in the IP group (p = 0.08) compared to the control group, but not for the EP group (Fig. 3D). No significant correlations were found between CVST score or age and within-module FC or SC connectivity.

#### Separate within-module connectivity

For FC, the strongest differences in within-modularity between patients and controls were found for module 4. Module 4 showed higher within module FC for the EP group (p = 0.012), while a trend was observed for the IP group (p = 0.071), compared to the control group (Fig. 3E). A positive association between CVST score (higher scores indicate reduced cognitive performance) and within module FC was found for module 4 in the control group (r = 0.36, p<0.04). The other modules did not reveal associations between CVST score and FC in neither the control group nor the EP group. Age was not significantly correlated with any of the within module FC values.

The within-module SC of module 4 was also significantly higher for the IP group (p = 0.013) compared to the control group. The other three modules did not show significant group differences in SC (Fig. 3F). For module 4 the within-module SC increased with higher CVST score (worse cognitive performance) in the EP group (r = 0.55, p<0.01). No association between SC and CVST score was found in any of the other modules. Age was not significantly correlated with any of the within-module SC values.

### Structure-function correlation

**Figure 4:**
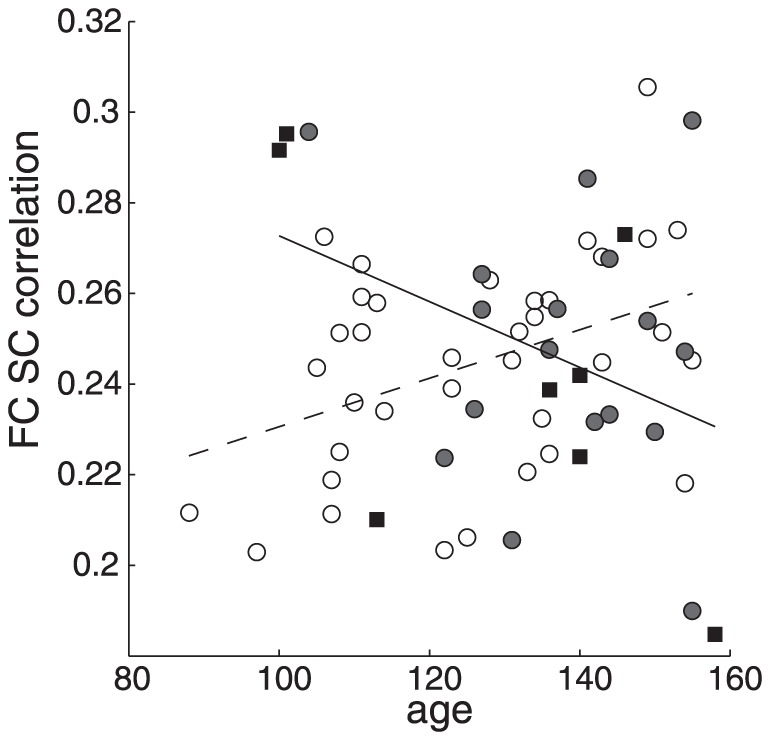
Function structure correlation with age. Coupling of SC and FC versus age of the control group (open circles) and unimpaired patient group (grey circles) and impaired patients (black squares). Each dot represents the correlation of all non-zero edge strengths of the FC and SC for that person. Regression lines for the control group (dashed) and entire patient group (solid) are shown. Note the increasing FC-SC correlation with age for the healthy controls, which was reversed for the patients with FLE.

The EP and IP groups did not differ significantly from the control group in SC-FC correlation. CVST score was not significantly associated with FC-SC coupling. A trend for a negative association was found in the EP group between FC-SC correlation and age (r = −0.38, p = 0.06), while for the control group a significant positive association (r = 0.40, p<0.01) was found (Fig. 4).

## Discussion

### Current findings

In this study we compared the FC and SC of cerebral networks in children with FLE to healthy controls and investigated whether the associated cognitive impairment in FLE is reflected by an aberrant functional or structural modular organization. For the whole brain network the functional network clustering, path length, and modularity appeared more sensitive than structural network measures to discern children with FLE, and particularly those with cognitive impairment from healthy controls.

The mean of both the cluster coefficient and the path length were higher in the patients and especially the cognitively impaired patients. Networks with high path length and high clustering may resemble towards regular networks (Sanz-Arigita EJ et al. 2010): these are networks with high local clustering but few connections linking distant nodes. These findings are in line with the modularity analysis, where an increased modularity score was found for the patients and especially the impaired patients: high path length and high clustering are signs that the patient networks are organized in tightly clustered modules with only limited inter-modular connectivity. For the modularity, patients, especially the cognitively impaired patients, showed higher scores than controls, suggesting the presence of more functionally isolated brain modules. It is possible that increases in coherent activity between functional systems (integration) might facilitate particular cognitive abilities. Therefore, a reduced amount of integration could lead to an impairment of cognitive functions. Previous studies have also found abnormal functional connectivity in children with FLE [34] as well as altered small world networks in other epilepsy patient groups [15], [49].

To focus on the organization of sub networks, modularity analysis resulted in a division of the whole brain network into four large-scale functional modules with relatively strong within-module and relatively weak between-module connections. The functional modular organization in childhood FLE appeared to be aberrant in the sense that between-module connectivity was weakest in the children with FLE who had cognitive impairments. In more detail it was found that module 4, which comprised large parts of the frontal and temporal lobe (see Figure S1 for a detailed view of this module), exhibited both increased functional and structural within-module connectivity relative to controls. For this particular module 4, the negative correlation between within-module FC and cognitive performance, as observed in healthy controls, was not present in the FLE patients. Also in this module, an increase in SC with decreased cognitive performance was observed for the FLE patients, but not for the controls.

### Global functional and structural network abnormalities

Whole brain functional network organization appeared to be disrupted in children with FLE, while the global structural network organization did not show such salient effects. Although structural network organization was not different in FLE patients compared to controls, stronger structural modularity was associated with worse cognitive scores in the patients. The deviant whole brain functional network measures were most pronounced in the cognitively impaired patients, and suggest that the functional network organization is linked to the cognitive pathology. Furthermore, the functional but not the structural abnormalities in network organization could imply that functional disturbances are more expressed or even precede structural abnormalities in childhood FLE. Further longitudinal research in developing children with epilepsy is required to investigate these hypotheses. Alternatively, differences in sensitivity of the different imaging modalities for detecting abnormalities could also underlie the observed results.

### Aberrant functional and structural modules

The stronger modular organization in children with FLE with cognitive impairment supports the hypothesis that whole brain connectional abnormalities can be traced back to differences in connectivity between and within more or less isolated functional modules [50], [51], [52], [53]. Between-module functional connectivity was decreased in the impaired children, while at the same time an increase in structural connectivity was observed. For both the functional and structural networks, an increase in within-module connectivity was found. Hence, children with FLE and cognitive impairments had an overall increase in structural connectivity, while the differences in functional connectivity were characterized by decreased between- and increased within-module connectivity.

Most striking for the relation between cognitive impairment and modular organization was the aberrant connectivity of module 4, which covers large parts of the frontal and temporal lobe. For this module, both the functional and structural within-module connectivity was increased, specifically for the cognitively impaired children, relative to the healthy controls. Considering the decline in higher cognitive functions for children with FLE, this observation seems to hint in more detail at the neuropathological substrate of cognitive impairment in childhood FLE.

### Developmental effect on connectivity

We found that the coupling between FC and SC increased with age in the healthy controls, which is in agreement with previous studies [24], [29], [54]. However, this increase was not found for the children with FLE. This could indicate that the normal development in SC-FC relation is disturbed in childhood FLE.

Some developmental processes such as an increase in white matter integrity or functional correlation with age might manifest in a gradual, brain wide manner and thus influence the overall mean SC or FC. Since the connectivity matrices were corrected for mean (whole brain) connectivity values, such effect might not be evident in the topological measures (L and C). Therefore, we also investigated the underlying data for the mean connectivity values. For the mean SC, an increase in FA and a decrease in ADC were observed with increasing age in the entire patient group, while FC was not correlated with age. Changes in FA and ADC with age have extensively been reported in literature [24], [55], [56] and are a sign of normal development. We could not detect significant group differences in mean FA or mean ADC, but a trend of larger relative tract volumes (thus SC values) in the impaired patient group was found. Given the narrow age range (8–13 year) of the subjects in this study for which correlations with age were found, studies with larger age ranges or preferably longitudinal studies are needed to infer on the expected abnormal developmental trajectories in childhood FLE.

### Limitations

The parcellation scheme used here was based on a subdivision of the Freesurfer cortical and subcortical region definitions. It has previously been shown that different parcellation schemes might yield different results [57], [58]. However, the fMRI data presented here was analyzed with a different parcellation scheme (Freesurfer default regions) which yielded similar results with respect to group differences in whole brain graph metrics [16]. We therefore are confident that our results are not specific to the parcellation scheme used.

Another point of interest might be to investigate the inter-individual differences in modular composition between subjects. Here, we used the group average to compute the modular composition to assure that the classification of edges as inter- or intra-modular are the same for all participants. However, recent advances in network analysis have made it possible to address the issue of group differences between groups in a more principled way [59], [60].

Lastly, we omitted an analysis on the group differences for each edge (i.e. does a specific connection between regions differ between groups?) as our hypothesis was that the patient population would display a large heterogeneity in terms of the exact location of the tissue abnormalities, and that therefore these effect would not be detectable at the group level (taking into account statistical difficulties with testing a large number of edges). New methods are available however to cope with the statistical problems in such analyses, which might be of interest to future studies [61].

### Conclusion

In children with FLE, it was shown that the more isolated functional brain sub networks appear to function, the more cognitively impaired these children are. This observation can be interpreted such that cognitively impaired patients have a less efficient interregional transfer of information between functional sub networks. As this effect was not directly evident from structural connectivity measures, this raises the question whether functional changes precede structural changes. Future studies are prompted for with patients in a broader age range and a longitudinal study design that might clarify the relation between structural and functional abnormalities in relation to cognitive developmental abnormalities in more detail.

## Supporting Information

Figure S1 **A detailed view of module 4.**
(TIF)
