# Supplementary figures and images for: Functional and Structural Network Impairment in Childhood Frontal Lobe Epilepsy
Source: PLoS One. 2014 Mar 4;9(3):e90068. doi: 10.1371/journal.pone.0090068 (PMC3942412; doi:10.1371/journal.pone.0090068)

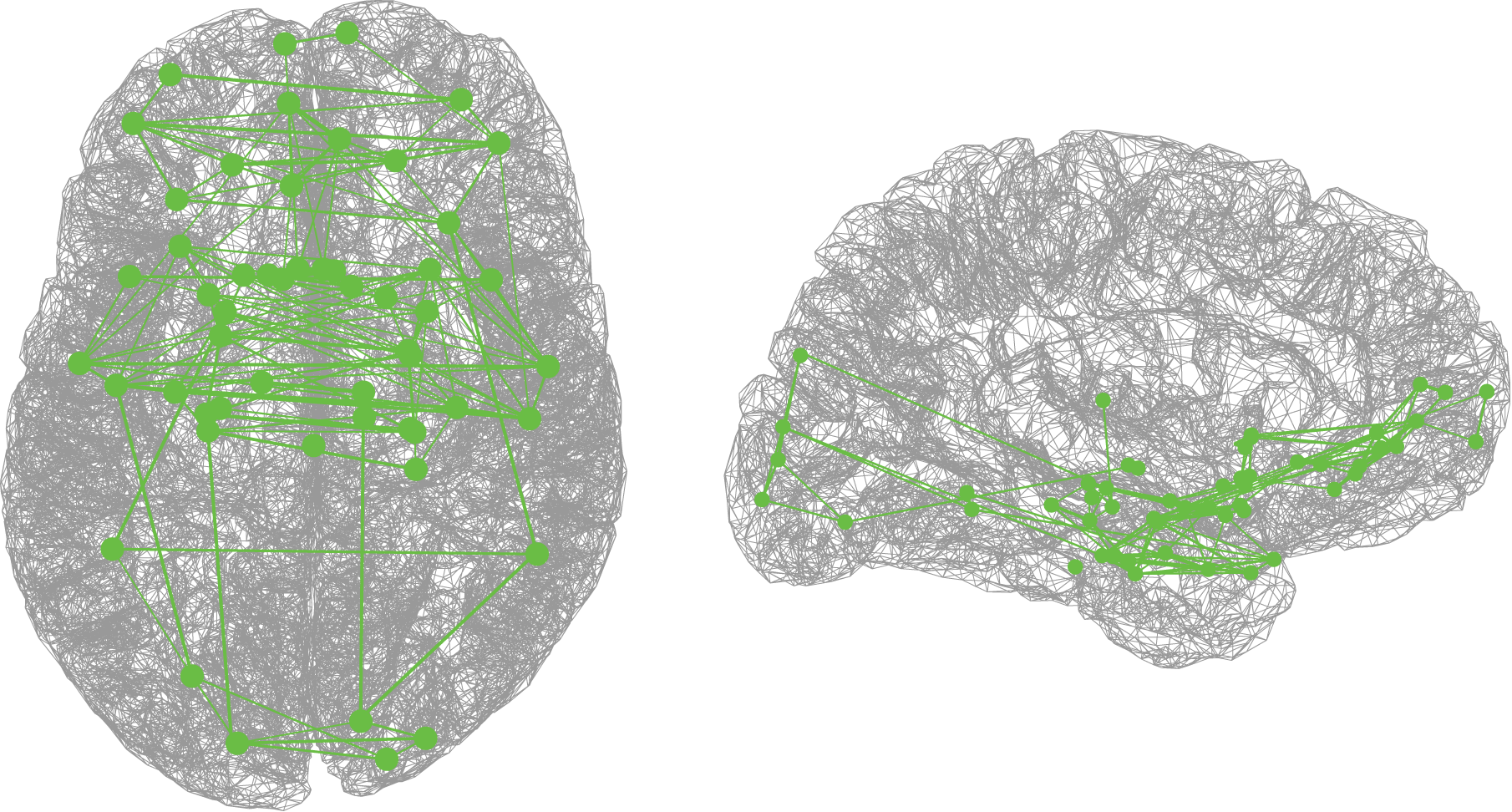

Supplement: Figure S1 — A detailed view of module 4. (TIF) [file pone.0090068.s001.tif]
